# Modern Advances in Velvet Antler Research: From Identification Methods to Pharmacological Mechanisms

**DOI:** 10.1155/ijfo/3848349

**Published:** 2026-04-25

**Authors:** Maodie Zhang, Xiaopeng Liu, Ning Jiang

**Affiliations:** ^1^ Hubei Key Laboratory of Biologic Resources Protection and Utilization, Hubei Minzu University, Enshi, 445000, China, hbmy.edu.cn; ^2^ School of Biological Science and Technology, Hubei Minzu University, Enshi, 445000, China, hbmy.edu.cn

**Keywords:** bioactivity, chemical composition, identification, pharmacological effects, traditional Chinese medicine, velvet antler

## Abstract

Velvet antler, a valued traditional medicine of animal origin, contains an abundance of bioactive compounds including amino acids, peptides, proteins, and nucleosides. It exhibits notable health‐promoting properties such as wound repair, antioxidant activity, and metabolic regulation, making it a high‐quality natural ingredient for functional foods and biopharmaceutical applications. Currently, velvet antler has gained widespread global use as a dietary supplement. Nevertheless, challenges remain in species authentication, mechanistic understanding of active constituents, and quality standardization, which impede the modern development and efficient utilization of this resource. This review summarizes recent advances in identification techniques for velvet antler—encompassing traditional, chemical, and molecular methods—analyzes its chemical composition and influencing factors, and explores the potential mechanisms behind its various pharmacological effects. Furthermore, key issues concerning safety evaluation, quality control standardization, and industry regulation are critically discussed. The findings provide a solid theoretical basis for improving the quality consistency and application reliability of velvet antler products, with the aim of promoting the scientific development and global industrialization of velvet antler resources in food science and biomedicine.

## 1. Introduction

Velvet antler is a prominent animal‐derived​ remedy in traditional Chinese medicine, traditionally classified among the “Three Great Tonics” together with ginseng and Ejiao. Its use dates back over 2000 years, with *Shennong’s Classic of Materia Medica* classifying it as a superior herb, emphasizing its functions in tonification, vitality enhancement, and longevity promotion [1]. With its unique nutritional and medicinal value, velvet antler has gained global recognition and formed a broad consumer market ranging from dietary supplements to functional food ingredients [2]. Countries such as the United States and New Zealand have also realized large‐scale production and commercialization of velvet antler products [2, 3].

The medicinal value of velvet antler arises from its complex mixture of bioactive constituents, including amino acids [4–6], peptides [7–9], proteins [10, 11], saccharides [12, 13], nucleosides [5, 14], and fatty acids [15], which collectively underlie its pharmacological properties. Notably, the chemical profile of velvet antler is dynamically influenced by multiple factors. As the antler matures, the content of active components gradually declines, leading to reduced quality [15]. Additionally, significant compositional variations exist along different sections of the antler: for example, crude fat, crude protein, sialic acid, uronic acid, and aminoglycans increase from the base to the tip, whereas collagen, ash, calcium, magnesium, and phosphorus show a decreasing trend [16]. This temporal and spatial heterogeneity not only complicates systematic research but also informs quality assessment criteria.

The complexity of velvet antler’s composition, coupled with market inconsistencies, has spurred advances in identification technologies. Modern chemical analysis [17, 18], molecular techniques [19–21], and multiomics approaches [22, 23] have substantially improved the scientific accuracy of authentication.

Well‐defined composition and reliable identification underpin pharmacological investigations. Emerging evidence suggests that velvet antler exerts diverse pharmacological activities through complex interactions between its bioactive constituents and cellular signaling networks. These biological effects encompass tissue regeneration [24, 25], oxidative stress regulation [26–28], metabolic homeostasis [29], and fibrotic modulation [30–32]. While substantial preclinical data exist, most studies have focused on isolated components or pathways, leaving the in vivo synergistic mechanisms, safety profiles, bioavailability, and clinical translation of active constituents insufficiently explored.

To our knowledge, existing reviews on velvet antler often overlook comprehensive safety evaluations and tend to focus narrowly on individual aspects—such as analytical techniques, chemical constituents, or pharmacological effects—rather than integrating identification methods, compositional features, mechanisms of action, safety assessment, quality control, and regulatory considerations into a cohesive analytical framework. This limitation hinders the translation of velvet antler from a traditional material into a modern, standardized product.

In light of these research gaps, the present review synthesizes recent advances to systematically examine the evolution and limitations of modern identification technologies, analyze the chemical characteristics and key determinants of velvet antler composition, elucidate the mechanisms underlying its diverse pharmacological effects, and assess current challenges related to safety, quality control, and global regulation. By establishing an integrated framework that links identification, composition, mechanism, safety, quality control, and supervision, this review aims to address the current lack of a holistic perspective and to provide a theoretical foundation for further scientific research and clinical application of velvet antler.

## 2. Identification Methods of Velvet Antler

Verifying the authenticity and species origin of velvet antler is essential for accurate comparison of its chemical composition and pharmacological effects. Reliable identification of source species, specification grade, and potential adulteration is required to ensure comparability and reproducibility in compositional analysis, biomarker screening, and efficacy assessment, thereby providing a sound basis for quality classification, mechanistic studies, and clinical or industrial use.

As a high‐value animal‐derived material used in both medicine and food, velvet antler is characterized by complex supply sources, substantial price variation, and long supply chains, all of which elevate the risks of adulteration, substitution, and mislabeling. According to the Chinese Pharmacopoeia (ChP), the only authentic sources of velvet antler are the unossified, velvet‐covered antlers of male *Cervus nippon* (sika deer) and *Cervus elaphus* (red deer). However, molecular evidence has revealed frequent species misidentification in commercial products. For example, a DNA barcoding study based on mitochondrial Cytochrome b (Cytb) found that only two of nine commercially labeled velvet antler samples matched their stated species, with the rest being counterfeit or mislabeled [33]. This indicates that species authentication remains a major gap in quality control.

Moreover, velvet antler is often marketed in processed forms such as slices, powder, or composite preparations. In such cases, traditional morphological identification becomes inadequate, underscoring the need to shift from reliance on “morphological experience” toward establishing robust “molecular evidence chains.”

### 2.1. Traditional Identification Techniques

The ChP primarily outlines traditional identification methods, specifically morphological and microscopic techniques. While these methods offer value for the preliminary screening of velvet antler, they are limited by low precision, difficulties with complex samples, and susceptibility to operator bias. For instance, morphological identification relies on visual inspection of physical characteristics [34], whereas microscopic identification examines cellular and tissue structures [35]. These approaches are effective for intact samples; however, their application is compromised when dealing with incomplete or deformed forms, such as powders and fragments.

For incomplete samples, the ChP also recommends chemical colorimetry and thin‐layer chromatography (TLC) based on active chemical components. Chemical colorimetry provides a preliminary authenticity assessment by detecting color reactions in proteins or amino acids. Despite some specificity, its sensitivity is easily compromised by impurities, potentially leading to false‐positive or false‐negative results. Similarly, TLC detects specific constituents such as glycine; however, the presence of such compounds alone is insufficient for definitive authentication. In summary, while microscopic identification, chemical reaction tests, and TLC are somewhat effective, they possess significant limitations, particularly for processed or complex samples. Consequently, improving the accuracy and reliability of velvet antler identification is imperative.

### 2.2. Chemical Analysis Techniques

In recent years, chemical analysis for velvet antler authentication and quality control has increasingly employed liquid chromatography–tandem mass spectrometry (LC‐MS/MS) combined with targeted quantification and chemometric methods. For example, a validated ultra‐performance liquid chromatography–tandem mass spectrometry (UPLC‐MS/MS) method using multiple reaction monitoring (MRM) enables rapid quantification of sphingolipid markers, supporting routine quality testing [17]. When chromatographic profiles appear visually similar, ultra‐performance liquid chromatography–quadrupole time‐of‐flight mass spectrometry (UPLC‐QTOF‐MS) peptide profiling of trypsin‐digested samples, coupled with principal component analysis (PCA) and orthogonal partial least squares discriminant analysis (OPLS‐DA), can effectively detect adulteration with reindeer velvet antler [18]. Compared to morphological and microscopic approaches, chemical analysis offers greater objectivity and is applicable to powdered samples, though it remains susceptible to interference from processing techniques.

### 2.3. Molecular Identification Techniques

Modern molecular biology techniques—including PCR, DNA barcoding, molecular markers, and multiomics—have substantially improved the identification and quality evaluation of velvet antler (Table 1).

**TABLE 1:** Overview of representative methods for velvet antler identification and quality authentication.

Authentication category	Key techniques	Detection threshold/performance	Advantages	Limitations	Refs
Traditional	Morphological identification	No quantitative LOD reported; used qualitatively for species/type and grade.	Very low cost, nondestructive, and rapid for crude antlers.	Strongly inspector‐dependent, not suitable for thin slices/powders, and cannot detect low‐level adulteration.	[34]
	Microscopic analysis	Qualitative only; no LOD or minimal adulteration proportion evaluated.	Provides more objective parameters than gross morphology and allows statistical clustering of species.	Still requires intact hairs, specialized training, and microscope and cannot be applied to decoctions, fine powders, or products where hairs are removed.	[35]

Chemical	Peptide‐based UPLC‐QTOF‐MS fingerprinting + PCA	Reported 100% correct classification of authentic vs. counterfeit samples in validation sets; explicit adulteration LOD not provided.	High chemical specificity; suitable for raw and processed products; allows simultaneous authentication and (when calibrated) semiquantitative quality assessment.	Requires expensive LC‐MS equipment, peptide libraries, and chemometric expertise; sensitive to digestion conditions and matrix effects; throughput moderate.	[18]
	Sphingolipids profiling (UPLC‐MS/MS)	LOD: 0.01–19.91 ng/mL	First systematic isolation of 2 novel and 6 known sphingolipids from *Cervus elaphus* velvet antler and development of a rapid, 7‐min MRM‐based UPLC‐MS/MS assay; high linearity (*R* ^2^ ≥ 0.996), good precision and recoveries, and suitable for routine quality control of *Cervus elaphus* velvet antler products.	Targeted panel limited to eight sphingolipids, so it cannot on its own distinguish deer species or complex adulteration patterns; requires high‐end LC‐MS/MS instrumentation and authentic standards.	[17]

Molecular	DNA barcoding/mini‐barcoding (Cytb + Cox1 + rrn12, COI)	No quantitative LOD was reported; studies demonstrate successful identification across commercial samples, but the minimal detectable adulterant proportion was not evaluated.	High species specificity; applicable to multiple sample forms (fresh, dried, powdered) and revealed frequent mislabeling and counterfeit deer species in commercial products.	Requires sequencing and reference databases; relatively time‐ and cost‐intensive; mainly qualitative, without resolving low‐level admixture or subspecies.	[33, 36]
	DNA mini‐barcode (COI) + HRM analysis	Template‐DNA detection limit 1 ng·μL^−1^; adulteration experiments showed reliable discrimination when ≥ 1% nonmedicinal deer was mixed into medicinal deer powders.	Short amplicons combined with HRM give rapid, closed‐tube species discrimination; tolerate degraded DNA; and can detect mixed (adulterated) deer powders in processed products.	Needs HRM‐capable qPCR instruments and reference melting profiles; mainly provides qualitative or semiquantitative assessment, with limited validation below a few percent adulteration.	[19]
	EST‐SSR markers	Not reported; designed for individual genotyping, not validated for quantitative adulteration detection.	High discriminatory power and transferability across several deer species; useful for population genetics and potential traceability.	Requires capillary electrophoresis and has not yet been applied directly to commercial velvet‐antler products.	[20]
	PCR‐based lateral‐flow strip (PCR‐LFB)	1 pg/μL for *Cervus nippon* DNA	High sensitivity, fast, low‐cost, specific, and detects trace adulteration.	DNA integrity‐dependent, subjective color interpretation.	[21]
	Triplex real‐time PCR	The LOD for velvet antlers of *Cervus nippon* and *Cervus elaphus* are 0.0001–0.000025 ng and 0.0025–0.00025 ng, respectively; the method can detect adulteration as low as 0.1% (w/w).	Rapid, high‐throughput, quantitative assay that can simultaneously identify and quantify *C. nippon* and *C. elaphus* DNA in complex products; validated on binary mixtures.	Needs real‐time PCR instrumentation, careful primer/probe design, and reference standards; only a limited set of species and mixtures was tested.	[37]

Omics	DIA‐based quantitative proteomics (LC‐MS/MS + DIA)	Not reported; relative‐abundance patterns only, no adulteration‐series LOD.	Provides high‐coverage protein fingerprints and growth‐stage‐specific markers; supports mechanistic understanding and potential quality markers.	Needs advanced LC‐MS/MS and bioinformatics and is still at the discovery stage without routine QC workflows.	[23]
	Untargeted metabolomics (UPLC‐MS/MS)	Not reported; exploratory comparison of pure species, without defined mixture detection limits.	High‐throughput coverage of amino acids, lipids, and other metabolites reveals 43 differential metabolites.	Limited power for strict species discrimination; requires UPLC‐MS/MS and careful control of diet/environment.	[22]

PCR and DNA barcoding rely on detecting specific gene‐region DNA sequences to differentiate deer species. By comparing results against reference databases, they can verify species authenticity and origin. PCR enables accurate identification even from minute, degraded, or contaminated samples, with novel triple real‐time PCR achieving detection limits as low as 0.1% (w/w) [37]. Gao et al. [21] developed a PCR‐based lateral flow biosensor (PCR‐LFB) for rapid visual authentication of dried *C. nippon* velvet products, with a detection limit of 1 pg/μL DNA. However, the method depends on DNA integrity and involves some subjectivity in colorimetric interpretation. Compared to real‐time quantitative PCR (qPCR), DNA barcoding is more cost‐effective and offers high specificity and stability, though its performance is limited by DNA extraction quality [36]. Zeng et al. [33] integrated DNA barcoding with stable isotope analysis and chemometrics to achieve a multidimensional quality and authenticity assessment through data clustering, highlighting the potential of combining DNA‐based and physicochemical indicators. Advances in sequencing have also enabled high‐resolution melting (HRM) analysis, which exploits base‐pair variations in target DNA for species discrimination. Feng et al. [19] established a cytochrome Oxidase I (COI)–based HRM method that successfully distinguishes *C. nippon* from *C. elaphus*, offering improved accuracy over traditional DNA barcoding.

Molecular markers provide another approach for systematic typing. Hsiao et al. [20] pioneered the use of expression sequence tag–simple sequence repeat (EST‐SSR) markers in cervids, developing 16 EST‐SSR markers capable of species identification and hybrid detection across Cervus species. SSR technology offers high polymorphism, reproducibility, and scalability, complementing mitochondrial gene‐based identification. Future integration of SNP chips and whole‐genome resequencing may further enhance resolution.

Multiomics technologies—such as proteomics and untargeted metabolomics—allow identification of potential quality markers and fingerprinting within complex velvet antler samples. By analyzing differential proteins or metabolites and pathway enrichment, these approaches can distinguish molecular profiles among velvet antler materials [22, 23]. Compared to experience‐dependent morphological identification, nonspecific physicochemical indicators, and the challenges of DNA extraction from processed products, multiomics strikes a better balance between precise identification and quality control. Nevertheless, the high costs associated with these technologies currently limit their suitability for routine, rapid, and low‐cost regulatory applications.

## 3. Chemical Constituents of Velvet Antler

The chemical profile of velvet antler closely resembles that of bone, primarily consisting of organic and inorganic components. Significant differences exist in both *C. nippon* and *C. elaphus* velvet antlers in key chemical constituents, including proteins [38], amino acids [4, 16], and inorganic elements [39]. The chemical composition of velvet antlers varies not only across different species but also among different anatomical regions within the same antler [40]. Interestingly, even within a single deer, the chemical composition of the antler tip may differ from that of the base [4, 16], potentially due to variations in nutrient absorption and metabolic activity during antler growth. These subtle compositional differences have a considerable impact on the pharmacological properties, quality assessment, and processing applications of velvet antler. Therefore, in‐depth research on its chemical constituents is essential for understanding its medicinal potential and ensuring the scientific management and sustainable utilization of velvet antler resources.

The chemical composition of velvet antler extracts is influenced by solvent polarity and extraction conditions, as variations in solvent polarity and extraction parameters lead to differences in the solubility of chemical components, which, in turn, affect the composition, color, texture, and antioxidant activity of the extracts [41]. Additionally, different processing techniques, including physical and chemical treatments [42, 43], affect the extraction efficiency, bioavailability, and stability of both water‐ and lipid‐soluble constituents, altering the therapeutic performance and safety profile of velvet antler–derived products.

Furthermore, the chemical composition of velvet antler undergoes dynamic changes depending on its growth stage and seasonal variations [44, 45]. The study indicates that as the antler growth cycle progresses, overall yield increases. However, the levels of bioactive compounds such as proteins, total amino acids, glycosaminoglycans, uronic acid, and sialic acid tend to decrease, while the content of ash and collagen increases [39]. These changes suggest that prolonged growth periods may lead to a reduction in biologically active substances, potentially affecting the overall quality of the antler. Understanding these variations is crucial for optimizing the harvesting period to maximize its medicinal potential.

### 3.1. Organic Components

The unique medicinal properties of velvet antler are primarily derived from its rich organic constituents, among which amino acids represent the most abundant nutritional components, including all eight essential amino acids that cannot be synthesized by the human body [6]. Studies have confirmed that antioxidant peptides containing aromatic amino acid residues, such as tryptophan and tyrosine, can effectively stabilize reactive oxygen species (ROS) through direct electron transfer, demonstrating strong antioxidant activity [46]. This is consistent with the findings of Ding et al. [47], who reported significant antioxidant activity in a purified velvet antler tetrapeptide, Trp‐Asp‐Val‐Lys.

Moreover, amino acid metabolism is essential for maintaining physiological functions, as these compounds participate in protein synthesis and degradation, energy production, and various metabolic pathways [48]. Studies have shown that glutamic acid present in water extracts of velvet antler plays a role in key metabolic pathways related to immune regulation [5], highlighting the potential of velvet antler as an immunomodulatory nutritional supplement. However, current research has largely remained at the level of pathway correlations, with limited exploration of the specific targets of glutamate or its synergistic interactions with other velvet antler components.

Notably, recent cutting‐edge research suggests that glutamate may represent an emerging strategy for the treatment of schizophrenia [49]. Nevertheless, the neuroregulatory effects of glutamate derived from velvet antler have not yet been reported, and its therapeutic potential remains underexplored. Future studies should integrate chemical composition analysis with mechanistic investigations to further elucidate the functional roles of amino acids in velvet antler, particularly glutamate, thereby providing a theoretical basis for expanding the medicinal applications of velvet antler resources.

Peptides are the principal bioactive compounds in velvet antler and exhibit a broad range of pharmacological effects [50]. The remarkable regenerative capacity of velvet antler is closely associated with peptide growth factors. Research indicates that velvet antler contains various such factors, including insulin‐like growth factor (IGF) [51], transforming growth factor (TGF) [52], thymosin β10 [53], and epidermal growth factor (EGF) [54], which play crucial regulatory roles in cell proliferation, differentiation, and tissue repair. Modern pharmacological studies suggest that TGF is closely involved in promoting wound healing and inhibiting scar formation [55–57], while fibroblast growth Factor 2 (FGF‐2) derived from velvet antler exhibits significant proangiogenic activity [58] and participates in protein synthesis and tissue regeneration [59]. Furthermore, deer‐derived IGF‐1 has been shown to alleviate IL‐1β‐induced chondrocyte inflammation and extracellular matrix (ECM) degradation in vitro, demonstrating potential antiosteoarthritic effects [60]. However, current research mainly focuses on functional validation of individual factors, with limited understanding of their synergistic effects, stability, and in vivo bioavailability. Systematic compositional analysis and mechanistic studies are still needed to support their pharmaceutical and functional development. Additionally, most existing research remains at the preclinical stage, and the actual efficacy of these peptides in complex physiological environments requires further validation, which also defines key directions for future research.

Beyond amino acids and peptides, proteins and polysaccharides are also critical bioactive macromolecular components in velvet antler. Studies have revealed that velvet antler contains various proteins such as collagen, serum transferrin, and apolipoprotein [61], and related extracts are associated with biological effects including anti‐inflammatory, antioxidant, and hepatoprotective activities in vitro and in animal models [10]. However, existing evidence is largely based on crude proteins or unrefined extracts, and the specific active components and their underlying mechanisms remain unclear. Meanwhile, polysaccharides from velvet antler have shown potential in modulating inflammation and improving lipid metabolism [13]. However, related studies mostly focus on crude polysaccharides, with systematic analyses of their structural characteristics and structure–activity relationships still lacking. Overall, research on velvet antler proteins and polysaccharides remains limited by the complexity of their composition and insufficient fine characterization. Future efforts should aim to clarify their active material basis through separation, purification, and structural identification, thereby providing a scientific foundation for their application in biopharmaceuticals and functional foods.

Velvet antler also contains various bioactive substances such as fatty acids and nucleosides. A recent study demonstrated that key bioactive fatty acids in velvet antler, including stearic acid (C18:0), linoleic acid (C18:2n), and arachidonic acid (C20:4n), promote healthspan and exert neuroprotective effects in Caenorhabditis elegans [62]. However, this research primarily relies on lower‐organism models; thus, the underlying molecular mechanisms and translational relevance in mammals require further validation. In recent years, nucleoside components have attracted increasing attention. Zhang et al. [14] indicated that nucleoside content can serve as a reference indicator for quality evaluation across different sections of velvet antler and exhibits antifatigue effects in vivo. Cross‐species metabolomic analysis further revealed that uridine, a nucleoside with regenerative potential, is pivotal to velvet antler stem cell and tissue regeneration [63]. Moreover, uridine from velvet antler can inhibit microglial glycolysis and M1 polarization via the HSP90/HIF‐1*α* pathway, thereby improving cognitive dysfunction in Alzheimer’s disease (AD) mouse models [64]. However, systematic validation of these mechanisms is still lacking, and their safety and applicability require further assessment. Future research should integrate quantitative component analysis, mechanistic elucidation, and multimodel validation to advance the scientific development of velvet antler–derived fatty acids and nucleosides.

In addition to the aforementioned components, velvet antler also contains trace amounts of fat‐soluble vitamins [15] (such as Vitamins A and E) and B vitamins [65] (B1, B2, and B6). Although current reports on the independent biological activities of velvet antler–derived vitamins remain limited, their potential value as cofactors for biological enzymes should not be overlooked. From a nutritional perspective, these trace components may function as cofactors, synergizing with primary constituents (e.g., bioactive peptides and polysaccharides) in velvet antler. For instance, Vitamin E detected in velvet antler acts as a natural free radical scavenger, potentially providing auxiliary support in alleviating exercise‐induced oxidative stress. Therefore, future research should extend beyond the identification of individual components. By integrating precision separation techniques with synergistic effect models, we can further explore the complementary contributions of these trace vitamins within the complex bioactive matrix of velvet antler. This approach will not only help comprehensively map the nutritional value of velvet antler as a functional food ingredient but also provide new perspectives for elucidating its multicomponent, multitarget pharmacological mechanisms.

### 3.2. Inorganic Components

The inorganic element content of velvet antler gradually increases from the tip to the base [39]. Velvet antler contains a variety of mineral elements, including major elements such as calcium (Ca), phosphorus (P), magnesium (Mg), potassium (K), and sodium (Na), as well as trace elements including aluminum (Al), zinc (Zn), manganese (Mn), iron (Fe), barium (Ba), nickel (Ni), and chromium (Cr) [40, 66]. The growth of velvet antler requires a substantial supply of minerals, particularly calcium and phosphorus, which are obtained both through dietary intake and mobilized from the deer’s bones. This process is associated with reversible osteoporosis in the skeletal system [67]. Significant differences in mineral composition exist between the base and tip of the antler. Specifically, the base contains higher levels of calcium and phosphorus, while potassium, zinc, and iron are relatively lower. In contrast, sodium and magnesium levels show no significant differences between these regions [68].

## 4. Multifunctional Pharmacological Activities and Underlying Mechanisms of Velvet Antler

**FIGURE 1:**
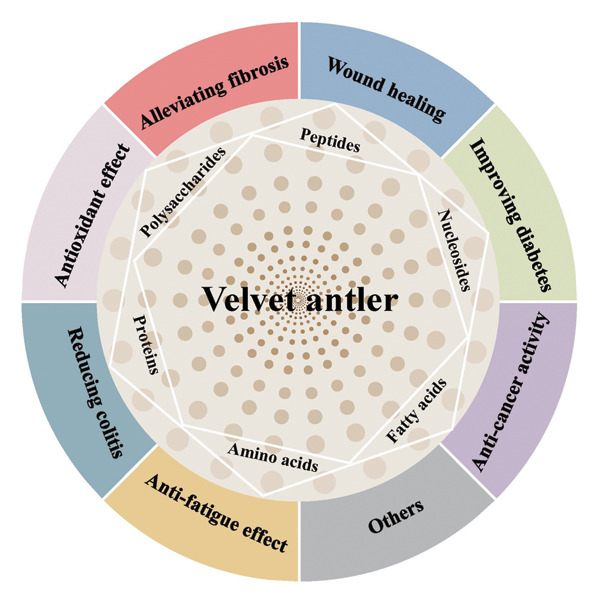
Schematic of the pharmacological action of velvet antler and its derivatives.

The rich spectrum of bioactive constituents found in velvet antler serves as the fundamental basis for its wide‐ranging pharmacological effects across multiple functional domains (Figure 1).

### 4.1. Wound‐Healing Activity of Velvet Antler

The process of tissue regeneration during wound healing is governed by intricate interactions between internal and external stimuli. It can be broadly divided into four sequential phases: hemostasis and coagulation, inflammation, proliferation, and remodeling. Each phase is characterized by the coordinated interaction of specific cell types and biochemical events [69].

Hemostasis and coagulation occur immediately upon injury to prevent blood loss and establish a provisional matrix that facilitates cellular infiltration and supports healing [69, 70]. Following this, the inflammatory phase ensues, during which neutrophils and macrophages are recruited to the wound site to clear bacteria and debris [71]. The proliferative phase then commences, characterized by the proliferation of fibroblasts, epithelial cells, and vascular endothelial cells. These cells work together to close the wound via ECM deposition, angiogenesis, and re‐epithelialization, ultimately forming granulation tissue [72].

Fibroblasts play a pivotal role in wound healing, a process that is essential for enhancing wound strength [73]. After the proliferation phase, the wound enters the remodeling phase, during which excess ECM is degraded by matrix metalloproteinases (MMPs), and collagen gradually transitions from Type III to Type I [25]. During this phase, scar tissue undergoes dynamic remodeling aimed at restoring the structural integrity and functional capacity of the skin [69]. Notably, the remodeling process is modulated by mechanical factors such as skin tension and pressure, which can contribute to the development of hypertrophic scars and keloids [25].

Wound healing is not solely governed by local factors but is also modulated by systemic conditions. For example, malnutrition, metabolic disorders (e.g., diabetes), advanced age, and infections can significantly impair the efficiency and quality of healing [73]. Furthermore, an imbalance in growth factor signaling may disrupt normal healing processes, whereas exogenous growth factor administration has been shown to enhance healing outcomes in both experimental and clinical settings [74].

**FIGURE 2:**
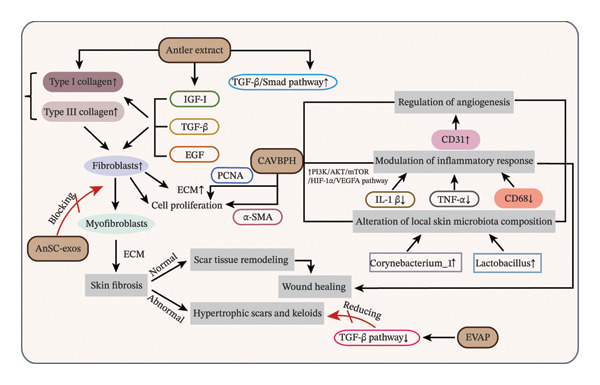
Biological basis of the wound‐healing and antiscarring effects of velvet antler and its derivatives. *Source:* [24, 55–57, 75].

Velvet antler and its derivatives have shown significant efficacy in promoting wound healing and minimizing scar formation, with mechanisms that involve the modulation of multiple biological processes and signaling pathways (Figure 2). During tissue repair, fibroblasts exhibit high plasticity and fulfill diverse functions at different stages, including promoting cell proliferation, facilitating migration, secreting cytokines and ECM, remodeling ECM, and participating in phagocytosis [76, 77]. The regulation of the local microenvironment (e.g., growth factors and inflammatory mediators) plays a critical role in determining fibroblast function and fate, thereby influencing their involvement in both physiological and pathological tissue repair [77].

Hao et al. [24] were the first to employ a chitosan/sodium alginate (CS/SA) hydrogel scaffold loaded with antioxidant short‐chain velvet antler blood peptides (VBP) to evaluate their effect on skin wound healing in Type 2 diabetes. They demonstrated that the resulting composite (CS/SA/VBP hydrogel, CAVBPH) markedly accelerated wound closure in a T2D mouse model. This effect was mediated through activation of the PI3K/AKT/mTOR/HIF‐1*α*/VEGFA pathway, significant downregulation of CD68, and reversal of inflammatory cytokine (TNF‐α and IL‐1β) expression, thereby modulating angiogenesis, inflammation, and the local skin microbiota. The multimodal action of CAVBPH not only enhanced cell proliferation but also improved overall repair outcomes via microbiota modulation. However, the precise biological mechanisms through which CAVBPH improves diabetic wound healing via the skin microbiota–active protein axis remain to be clarified.

Collagen synthesis and degradation are pivotal in wound‐healing. Fibroblasts exhibit strong secretory activity, producing various ECM components such as collagen and elastin, which are essential for maintaining tissue structure and function [78]. Velvet antler extracts are rich in peptide growth factors, including TGF‐β, IGF‐I, and EGF. In a rat model of full‐thickness skin wounds, these bioactive constituents were found to enhance fibroblast expansion and promote collagen production, ultimately facilitating the wound healing process [75]. Although these results suggest that velvet antler extracts could serve as a potential source of targeted growth‐factor therapies for wound healing, the precise synergistic or antagonistic interactions among these factors and their molecular mechanisms remain incompletely understood. Similarly, velvet antler protein extracts have been reported to enhance wound regeneration in full‐thickness excisional rat models by promoting fibroblast proliferation, accelerating Type I and Type III collagen synthesis, and activating the TGF‐β/Smad signaling pathway [57].

Scar formation presents a major challenge in wound‐healing, yet velvet antler peptides (VAPs) offer distinct advantages in addressing this issue. Velvet antler‐derived peptides (EVAP) significantly reduce the expression of TGF‐β1, Smad2, phosphorylated Smad2 (p‐Smad2), α‐smooth muscle actin (α‐SMA), and Type I collagen by suppressing the activation of the TGF‐β signaling pathway, thereby markedly attenuating scar formation and improving wound‐healing quality in a rat full‐thickness cutaneous wound model [55]. Additionally, exosomes derived from velvet antler stem cells (AnSC‐exos) have drawn increasing attention for their role in promoting regenerative healing. Zhang et al. [56] first demonstrated that local periwound injection of AnSC‐exos in a rat full‐thickness wound model downregulates TGF‐β1 signaling, inhibits the transdifferentiation of dermal fibroblasts into myofibroblasts, reduces wound contraction and collagen deposition, and ultimately facilitates regenerative wound healing. These findings underscore the potential of AnSC‐exos as a novel cell‐free therapeutic strategy for scarless skin repair.

### 4.2. Antioxidant Pharmacological Activities of Velvet Antler

Oxidative stress represents a central contributor to the pathogenesis of numerous chronic diseases. Bioactive compounds isolated from velvet antler have demonstrated considerable antioxidative potential, which is achieved through both direct radical‐scavenging activity and the modulation of endogenous antioxidant defense systems. These bioactivities support the potential development of velvet antler as a multifunctional ingredient in health‐promoting foods, nutraceutical formulations, and emerging biopharmaceutical products.

**FIGURE 3:**
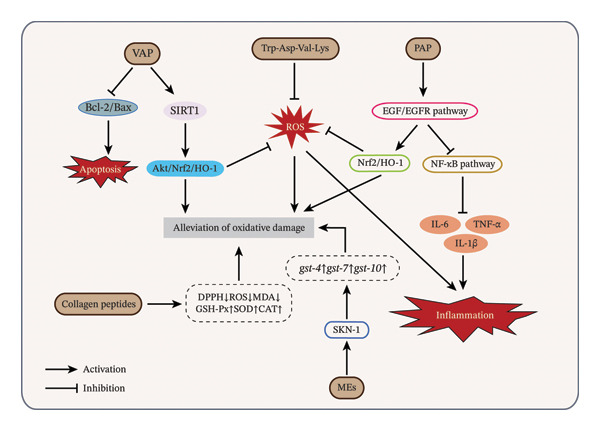
Velvet antler–mediated antioxidant activity: Key bioactive compounds and biological mechanisms. *Source:* [26, 27, 47, 79, 80].

The antioxidant activity of velvet antler involves multiple bioactive constituents and biological mechanisms (Figure 3), showing strong antioxidant effects in proteins, peptides, lipids, and alkaloids. Proteins and their enzymatic hydrolysates are recognized as critical contributors to these effects. Ding et al. [47] demonstrated that a purified velvet antler tetrapeptide (Trp‐Asp‐Val‐Lys, 547.29 Da) scavenges peroxyl radicals (IC_50_ = 0.028 mg/mL), inhibits ROS production, and protects against AAPH‐induced oxidative stress in hepatocytes and zebrafish models. Subsequent research indicated that VAPs not only exert direct antioxidant activity but also mitigate oxidative damage in PC12 cells by activating the SIRT1‐dependent Akt/Nrf2/HO‐1 pathway [26]. Furthermore, VAP attenuated dopaminergic neuron loss in a Parkinson’s disease mouse model and improved neurological health by modulating the gut microbiota.

Additionally, pilose antler peptide (PAP) has been shown to upregulate Nrf2/HO‐1 expression via the EGF/EGFR signaling pathway while suppressing NF‐κB signaling. This leads to increased superoxide dismutase (SOD) activity, reduced malondialdehyde (MDA) levels, and decreased release of proinflammatory cytokines such as IL‐1β, TNF‐α, and IL‐6, thereby protecting osteoblasts from oxidative stress–induced injury [27]. In a rat model of hypoxic‐ischemic (HI) injury, PAP alleviated brain damage by modulating the gut microbiota to enhance intestinal barrier function, which in turn reduced oxidative stress and inflammatory responses [28]. However, this study did not identify the specific active peptides within the PAP preparation or fully clarify the underlying molecular pathways involved.

Notably, antioxidant peptides derived from velvet antler also exhibit multifunctional properties in blood pressure regulation. Studies demonstrate that a purified pentapeptide, Asp‐Asn‐Arg‐Tyr‐Tyr (MW = 730.31 Da), isolated from the enzymatic hydrolysate of velvet antler not only exhibits potent antioxidant activity but also effectively lowers blood pressure in spontaneously hypertensive rats by inhibiting angiotensin‐converting enzyme (ACE) [81]. This pentapeptide binds to the active site of ACE, blocking its catalytic activity, which underscores the potential therapeutic value of velvet antler–derived antioxidant peptides in cardiovascular health [81].

The antioxidant capacity of velvet antler is primarily attributed to its nonprotein bioactive constituents. Collagen‐derived peptides isolated from velvet antler exhibit strong free radical–scavenging activity against 1,1‐diphenyl‐2‐picrylhydrazyl (DPPH). In addition, these peptides enhance the endogenous antioxidant defense system, as evidenced by increased activities of SOD, catalase (CAT), and glutathione peroxidase (GSH‐Px), along with a concomitant reduction in oxidative stress–related biomarkers, such as ROS and MDA. These properties suggest potential applications in skin cell protection [79]. Gelatin and its enzymatic hydrolysates from velvet antler also exhibit strong antioxidant activity across various assays. By enhancing thermal stability and interfacial properties, these compounds show increased potential for use in food and health products [82]. Furthermore, velvet antler methanol extracts (MEs) reduce endogenous ROS in *Caenorhabditis elegans* and upregulate the expression of antioxidant genes, including *gst-10*, *gst-7*, and *gst-4*, through activation of SKN‐1, thus extending the survival time of *C. elegans* under oxidative stress [80]. Notably, the antioxidant capacity of velvet antler may vary depending on species and extraction method. The 75% ethanol extract of Rusa velvet antler exhibits the strongest antioxidant activity in DPPH and ferric reducing antioxidant power (FRAP) assays compared to hexane and water extracts and is enriched in antioxidant fatty acids such as oleic acid, linoleic acid, and alpha‐linolenic acid [41].

In summary, the antioxidant properties of velvet antler are attributed to a variety of chemical constituents (such as peptides, phospholipids, fatty acids, and other compounds) and multiple biological pathways (including Nrf2/HO‐1, EGF/EGFR, and NF‐κB). These mechanisms collectively contribute to the attenuation of oxidative injury through multiple processes, including neutralization of reactive species, reinforcement of endogenous antioxidant enzyme systems, and regulation of redox‐related signaling pathways. Additionally, certain antioxidant peptides display other bioactivities, such as antihypertensive and neuroprotective effects. Despite these advances, the current understanding remains incomplete. Future studies should focus on optimizing extraction strategies, identifying and characterizing bioactive constituents, and elucidating the molecular basis underlying their antioxidant effects. These efforts will facilitate the identification of key functional components and targets, thereby promoting their applications in neuroprotection, antiaging interventions, and functional food development.

### 4.3. The Role of Velvet Antler in Diabetes Management and the Modulation of Glucose and Lipid Metabolism

Diabetes is a multifactorial metabolic disease marked by insulin resistance, impaired pancreatic β‐cell function, and persistent hyperglycemia, frequently accompanied by dyslipidemia and oxidative stress [83]. Natural products have recently attracted considerable attention as potential adjunctive agents in the management of diabetes. Among these, velvet antler, with its diverse bioactive components, has shown promising effects in reducing blood glucose, regulating lipid profiles, and exerting antioxidative. Studies suggest that velvet antler and its active peptides can modulate glucose and lipid metabolism through multiple pathways, enhance pancreatic function, and mitigate inflammatory responses, offering new possibilities for the prevention and management of diabetes and its associated complications.

Polypeptides derived from velvet antler have been shown to effectively reduce fasting blood glucose levels in diabetic mouse models, enhance glucose tolerance and insulin secretion, and improve the pathological state associated with diabetes. Notably, polypeptides from red deer antler (PRDA) exhibit a significant hypoglycemic effect in STZ‐induced diabetic mice by upregulating key enzymes involved in hepatic glucose metabolism, improving glucose metabolic efficiency, and reducing oxidative stress, thereby maintaining glycemic homeostasis [84]. Additionally, the antidiabetic peptide CPU2206 isolated from *C. nippon* velvet antler demonstrates similar hypoglycemic effects across various mouse diabetic models and exerts protective effects on pancreatic islet tissue [85]. In contrast to traditional hypoglycemic approaches, the conditioned medium of velvet antler stem cells (AnSC‐CM) enhances insulin secretion in Type 1 diabetic models by significantly inhibiting the NF‐κB signaling pathway in pancreatic and hepatic tissues [86].

In addition to its regulatory effects on glucose metabolism, velvet antler extract is critically involved in lipid metabolism. Diabetes is often associated with dyslipidemia, characterized by elevated triglyceride, elevated cholesterol, and low‐density lipoprotein cholesterol levels [84]. Active peptides derived from velvet antler have demonstrated significant efficacy in improving hyperlipidemia linked to diabetes. Studies show that treatment with PRDA and CPU2206 significantly reduces serum levels of triglycerides, free fatty acids, and total cholesterol in diabetic mice, indicating their potential to modulate lipid metabolic pathways, decrease lipid accumulation, and enhance insulin sensitivity [84, 85]. Furthermore, AnSC‐CM has been shown to regulate lipid metabolic abnormalities in diabetes by improving hepatic metabolic function and mitigating inflammatory damage [86]. These findings provide further support for the potential therapeutic value of velvet antler extract in managing disorders of glucose and lipid metabolism.

Oxidative stress responses play crucial roles in the pathogenesis and progression of diabetes. The anti‐inflammatory and antioxidant properties of velvet antler extract may be key mechanisms underlying its hypoglycemic and lipid‐lowering effects. Studies have shown that PRDA treatment significantly reduces MDA levels, a marker of oxidative stress, in both the serum and liver of diabetic mice. At the same time, the activities of CAT, SOD, and total antioxidant capacity (T‐AOC) were markedly increased, suggesting that PRDA alleviates oxidative damage by reducing ROS accumulation, thus improving the pathological process associated with diabetes [84]. Collectively, these findings indicate that the antioxidant properties of velvet antler extract may act synergistically in its therapeutic intervention for diabetes, providing critical biological evidence for enhancing pancreatic islet function, improving glucose and lipid metabolic disorders, and reducing diabetes‐related complications. Despite these promising findings, the precise intracellular cascades and molecular networks responsible for these biological effects demand deeper, more targeted investigation. Future research should focus on systematic component analysis and mechanism clarification to provide a solid foundation for its medicinal and functional development.

### 4.4. Anticancer Properties of Velvet Antler

**FIGURE 4:**
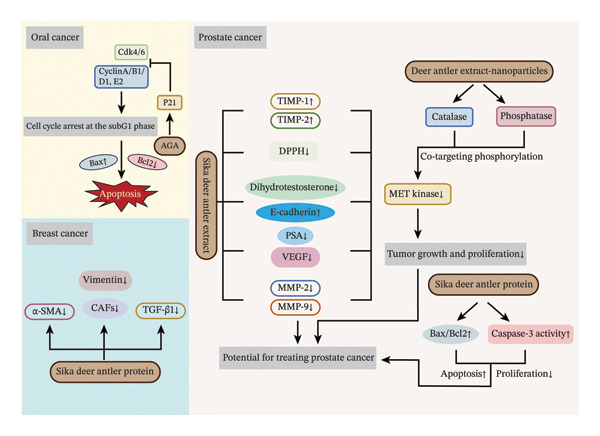
Anticancer properties of velvet antler. *Source:* [88–93].

Cancer, a major global public health challenge, represents a significant threat to human health due to its high incidence and mortality rates [87]. With an expanding understanding of the mechanisms driving cancer development and the continuous advances in therapeutic strategies, the discovery and development of novel anticancer drugs have become central to medical research. In this context, natural products, known for their unique chemical structures, biological activities, and relatively low toxicity, have emerged as vital sources for the development of new anticancer agents. The anticancer properties of velvet antler have been well‐documented, with studies demonstrating its efficacy against various tumor types through multiple biological mechanisms (Figure 4).

Previous studies have revealed that velvet antler extracts suppress tumor growth through multiple mechanisms, including the inhibition of malignant cell proliferation, the triggering of programmed cell death, the arrest of cell cycle progression, and the regulation of immune functions. Lu et al. [88] found that a compound herbal preparation (AGA) containing velvet antler extract (A), *Ganoderma lucidum* (G), and *Antrodia camphorata* (A) effectively suppresses the proliferation of oral cancer cells. At the mechanistic level, AGA downregulates the expression of cell cycle regulators (CDK4, CDK6, Cyclins A, B1, D1, and E2), upregulates the proapoptotic protein Bax, and suppresses Bcl‐2 expression, ultimately arresting cancer cells in the Sub‐G_1_ phase. Notably, these in vitro molecular effects were partially corroborated in a nude mouse xenograft model, where AGA significantly suppressed tumor growth without observable liver or kidney toxicity. However, the study primarily focused on phenotypic observations and key regulatory protein analyses, lacking deeper insight into direct molecular targets or receptor‐level mechanisms.

Also noteworthy is the broad‐spectrum anticancer effect of velvet antler extract. Studies indicate that deer velvet antler extract (DVA) exhibits significant antitumor activity across various tumor cell lines, including glioblastoma, colorectal cancer, breast cancer, and leukemia, reducing cell viability and inhibiting migratory capacity. Importantly, in a mouse glioblastoma xenograft model, intraperitoneal administration of DVA at 200 mg/kg for 28 consecutive days reduced tumor weight by 66.3%. Mechanistically, DVA appears to reduce tumor mass and induce liquefactive necrosis by upregulating immunomodulatory targets such as CD30L and CD40 while downregulating protumor factors such as FASL and CX3CL1, demonstrating in vivo anticancer efficacy [94]. These findings further suggest that velvet antler may exert anticancer effects not only through direct action on tumor cells but also via modulation of the host immune system. Nevertheless, identification of the specific bioactive molecules in DVA responsible for antitumor activity and elucidation of their underlying mechanisms remain essential for developing broad‐spectrum, low‐toxicity anticancer drugs.

The application of velvet antler in prostate cancer (PC) research is particularly significant. In vitro studies confirm that extracts from *C. nippon* velvet antler inhibit the migration of PC cells by suppressing prostate‐specific antigen (PSA) expression. Furthermore, these extracts downregulate MMP‐9 and vascular endothelial growth factor (VEGF), while upregulating tissue inhibitors of Metalloproteinases 1 and 2 (TIMP‐1 and TIMP‐2) [89]. However, the specific molecular mechanisms underlying this anti‐PC activity require further investigation. In vivo studies further validate its efficacy: Velvet antler extract significantly suppresses tumor growth in PC xenograft models by reducing PSA and dihydrotestosterone expression, achieving an inhibition rate of 65.08%. The extract also decreased VEGF, MMP‐2, and MMP‐9 levels, increased TIMP‐1 and TIMP‐2 expression, and upregulated E‐cadherin, thereby inhibiting epithelial‐mesenchymal transition (EMT)–related genes. Together, these effects attenuate PC tumor growth, migration, and invasion, demonstrating its potential as an anti‐PC agent [90].

At the nanoscale, velvet antler extract contains nanoparticles exhibiting nanozyme‐like activities, including phosphatase and CAT. These nanoparticles, in combination with MET kinase, suppress PC cell viability. Molecular docking and network pharmacology analyses further confirm that velvet antler extract targets MET kinase, indicating that combining velvet antler nanoparticles with MET kinase could offer a promising therapeutic strategy for PC [91]. However, the underlying mechanisms still require further analysis. Additionally, protein extract from velvet antler has been shown to exert antiproliferative and proapoptotic effects on PC cells, potentially through the upregulation of Caspase‐3 activity and an increased Bax/Bcl‐2 ratio. In mouse models, it has demonstrated high biocompatibility and safety [92]. Furthermore, the anticancer activity of velvet antler extract is closely linked to its antioxidant properties, which reduce gene mutations and DNA damage caused by oxidative stress via free radical scavenging and enhancement of the cellular antioxidant defense system [89]. This mechanism further supports the multitargeted effects of velvet antler extract in PC therapy.

Velvet antler extract offers unique advantages in triple‐negative breast cancer (TNBC) treatment. Its active component, water‐soluble velvet antler peptides (PAWPs), synergistically enhances chemotherapy efficacy. A study demonstrates that PAWPs increase TNBC sensitivity to neoadjuvant chemotherapy (NAC), inhibit tumor growth, and mitigate chemotherapy‐induced side effects such as weight loss and liver damage induced by chemotherapy in mice. Mechanistically, PAWPs target cancer‐associated fibroblasts, downregulating TGF‐β1, α‐SMA, and vimentin expression in tumor tissues. This weakens the immunosuppressive barrier and promotes the proliferation and infiltration of CD4^+^ and CD8^+^ T‐cells, thereby reversing chemotherapy‐induced immunosuppression [93]. These results indicate that velvet antler extract not only exhibits independent anticancer properties but also acts as an adjuvant sensitizer for chemotherapy, helping to mitigate immunosuppression and enhance tumor chemosensitivity.

### 4.5. The Therapeutic Potential of Velvet Antler in Colitis Intervention

**FIGURE 5:**
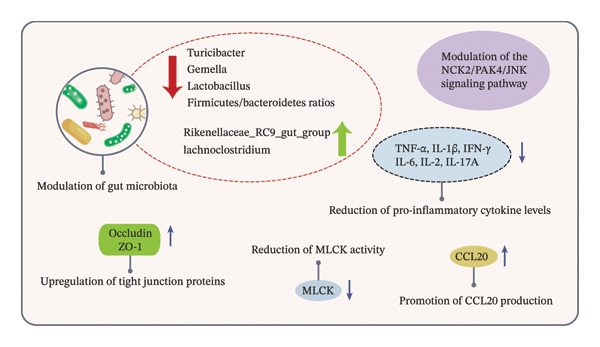
Modulation of colitis by velvet antler via multiple mechanisms. *Source:* [95–97].

Velvet antler has been shown to have the potential to regulate intestinal health and alleviate colitis symptoms. While its anti‐inflammatory effects are among its various pharmacological actions, this review focuses on how velvet antler can reduce colitis symptoms by modulating the balance of intestinal flora, enhancing intestinal barrier function, and influencing cell signaling pathways (Figure 5), thus providing new scientific evidence and research directions for colitis treatment. Huang et al. [95] first demonstrated that velvet antler water extract can target and repair the colonic epithelial barrier. In a dextran sulfate sodium (DSS)–induced colonic epithelial cell model (Caco‐2), the extract reduces transepithelial electrical resistance values and the permeability of large molecules, thereby reversing abnormal intestinal permeability. The potential molecular mechanisms involve, on one hand, upregulation of tight junction proteins (occludin and tight junction Protein 1, ZO‐1), and on the other hand, reduction of barrier damage caused by cytoskeletal contraction through decreased myosin light chain kinase (MLCK) activity. Additionally, the induced production of C‐C chemokine Ligand 20 (CCL20) may further help stabilize intestinal barrier function by recruiting cells involved in epithelial repair. However, the complete mechanistic pathway remains to be fully elucidated.

The aforementioned in vitro observations have been corroborated in vivo. Huang et al. [96] demonstrated that the water extract of *C. elaphus* velvet antler effectively alleviated symptoms in a DSS‐induced colitis mouse model, as reflected by reduced inflammatory infiltration, diminished crypt damage, decreased goblet cell depletion, and improved tissue architecture. The underlying mechanism appears to involve modulation of systemic immune responses through significant reduction of Th1‐associated proinflammatory cytokines in the spleen and serum, such as TNF‐α, IL‐1β, and IFN‐γ. Notably, high‐dose water extract of *C. elaphus* velvet antler exhibited more pronounced inhibitory effects on IL‐6, IL‐2, and IL‐17A. Furthermore, treatment with this water extract was associated with elevated levels of intestinal tight junction proteins, including occludin and zonula Occludens 1 (ZO‐1), which contributed to improved epithelial barrier function and junctional stability.

Velvet antler extract has been shown to modulate the intestinal microbiota, alleviating DSS‐induced dysbiosis and supporting the recovery of beneficial microbial communities. Research has shown that water extracts of *C. elaphus* velvet antler can activate the hypoxia‐inducible Factor‐1*α* (HIF‐1*α*) signaling pathway while modulating gut microbial metabolic outputs, leading to increased levels of metabolites such as acetate and propionate and ultimately contributing to the alleviation of colitis [96].

Concurrent microbiome analysis confirmed that velvet antler extract reduces the abundance of proinflammatory bacteria such as Turicibacter and Gemella, while promoting the growth of anti‐inflammatory taxa including *Rikenellaceae*_RC9_gut_group and *Lachnoclostridium*, indicating that its anticolitis effects may be mediated in part via modulation of gut microbiota composition [96].

In a comparative study, Li et al. [97] demonstrated that vinegar‐treated deer antler (VCCD) outperformed water‐treated deer antler (WCCD) in improving spleen–kidney yang deficiency‐type ulcerative colitis. VCCD achieved this by optimizing the gut microbiota—increasing the Bacteroidetes/Firmicutes ratio and the abundance of *Lactobacillus*—and targeting the NCK2/PAK4/JNK signaling pathway to suppress colonic inflammatory responses. Through the combined reduction of the colonic mucosal damage index (CMDI) and modulation of microenvironmental signaling axes, these findings provide contemporary scientific support for the value of traditional velvet antler processing methods.

In summary, velvet antler extract has demonstrated significant protective effects in alleviating colitis symptoms, enhancing intestinal barrier function, modulating immune‐inflammatory responses, and restoring intestinal microbiota homeostasis. These findings highlight that velvet antler extract may be a promising therapeutic candidate for inflammatory bowel disease (IBD). Moving forward, identifying the exact bioactive fractions and detailing their molecular interactions will be critical for translating these discoveries into advanced functional ingredients and targeted therapies for gut pathologies.

### 4.6. Antifatigue Effects of Velvet Antler: Muscle Modulation, Antioxidative Mechanisms, and Characterization of Active Components

**FIGURE 6:**
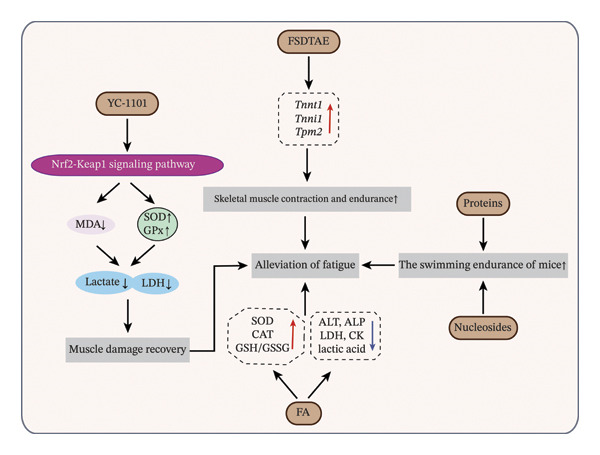
Mechanistic insights into the antifatigue potential of velvet antler: A multifactorial regulation and bioactive synergy. *Source:* [14, 31, 32, 40, 98].

With the accelerating pace of modern life and increasing occupational stress, prolonged high‐intensity workloads and unhealthy lifestyle habits have contributed to physical fatigue, making it a significant health concern [30]. As a traditional Chinese medicinal material, velvet antler is increasingly recognized for its potential antifatigue properties. It exerts multidimensional effects through mechanisms involving muscle function modulation, oxidative stress attenuation, and the coordinated actions of multiple bioactive constituents (Figure 6).

From the perspective of muscle physiology, velvet antler extract demonstrates dual benefits of enhancing muscle contractility and promoting tissue repair. Park et al. [31] demonstrated that enzymatically hydrolyzed deer antler extract (YC‐1101) enhances antioxidant defense via the Nrf2‐Keap1 signaling pathway, promotes C2C12 myoblast proliferation and muscle regeneration, and extends swimming duration in a forced swimming model, indicating its efficacy in mitigating fatigue. Similarly, Chen et al. [32] reported that Formosan sambar deer antler extract (FSDTAE) enhances skeletal muscle contraction and endurance by upregulating genes such as *Tnnt1*, *Tnni1,* and *Tpm2*, which are crucial for muscle contraction dynamics.

Additionally, alleviating exercise‐induced oxidative stress damage represents another core pathway through which velvet antler exhibits its antifatigue potential. Jeon et al. [98] revealed that *Lactobacillus curvatus* HY7602‐fermented velvet antler extract (FA) significantly alleviates physical fatigue in mice by reducing H_2_O_2_‐induced cytotoxicity and ROS levels, enhancing antioxidant enzyme activity, improving the glutathione/oxidized glutathione (GSH/GSSG) ratio, and upregulating hepatic antioxidant gene expression.

Crucially, the antifatigue effects of velvet antler exhibit significant substance dependence, with its efficacy strongly correlated to the spatial distribution patterns of nucleosides and protein‐based components. From tip to base, velvet antler is sequentially divided into four distinct regions: wax layer, powder layer, gauze layer, and bone layer [40]. Zhang et al. [14] identified a strong positive correlation between nucleoside‐rich fractions in velvet antler wax slices and prolonged swimming endurance, suggesting that nucleosides may be key bioactive compounds contributing to antifatigue activity. Similarly, Chen et al. [40] demonstrated that velvet antler wax slices significantly enhance swimming endurance in mice and established a highly significant correlation (*r* = 0.997) between antler‐derived proteins and antifatigue effects.

While these findings highlight the therapeutic potential of velvet antler in fatigue management, the precise core bioactive compounds, molecular targets, regulatory pathways, and the impact of different processing techniques remain insufficiently characterized. Future research should aim to elucidate the complex antifatigue mechanisms of velvet antler to support its development as a scientifically validated functional food ingredient or therapeutic candidate for fatigue‐related disorders.

### 4.7. Application Potential of Velvet Antler in Organ Fibrosis Therapy

Organ fibrosis is a pathological process triggered by various factors, including chronic inflammation, autoimmune diseases, metabolic disorders, drug‐ or toxin‐induced damage, ischemia or hypoxia, infections, and complex interactions between genetic and environmental factors, as well as tumor progression [99]. These factors collectively lead to the replacement of normal tissue architecture with aberrantly proliferating fibrotic tissue, ultimately impairing organ function. Virtually, all organs are susceptible to fibrosis [100].

In the context of liver fibrosis, a thymosin β10 (Tβ‐10) rich in velvet antler stem cell–derived peptides (AnSC‐P) has been shown to attenuate fibrosis by inhibiting the TGF‐β1/SMAD signaling pathway [101]. A recent study demonstrated that in a CCl_4_‐induced mouse model of liver fibrosis, treatment with Tβ‐10 significantly reduced serum markers of liver injury, including aspartate aminotransferase, alanine aminotransferase, and total bilirubin, while concurrently increasing albumin levels, suggesting a protective effect against liver damage. Moreover, in vitro assays using hepatic stellate cell (HSC) revealed that Tβ‐10 effectively inhibited TGF‐β1‐induced activation, significantly reducing the expression of α‐SMA and Type I collagen. These findings suggest that Tβ‐10 exerts its antifibrotic effects by modulating key signaling pathways involved in liver fibrosis [101].

In pulmonary fibrosis research, AnSC‐Exos have been shown to effectively mitigate bleomycin‐induced lung fibrosis in mice, primarily by inhibiting monocyte‐macrophage recruitment, thereby reducing the release of profibrotic factors [102]. Treatment with AnSC‐Exos significantly decreased the deposition of Type I and Type III collagen in lung tissue while reducing the activation of pulmonary fibroblasts, suggesting that AnSC‐Exos may exert antifibrotic effects by regulating the dynamic balance of the ECM. Additionally, exosomal miRNAs let‐7b and let‐7a were found to downregulate CCL7 expression in fibroblasts, thereby limiting M2 macrophage accumulation and further suppressing pulmonary fibrosis progression [102].

In the context of cardiac fibrosis, the velvet antler peptide sVAP32 has been reported to attenuate myocardial fibrosis in Sprague–Dawley (SD) rats induced by transverse aortic constriction by blocking the TGF‐β1 signaling pathway [103]. Mechanistically, sVAP32 prevents TGF‐β1 from binding to its receptor, thereby reducing the phosphorylation of downstream Smad2/3 and ERK1/2, ultimately inhibiting fibroblast activation and excessive ECM deposition in SD rat cardiac tissue. Moreover, sVAP32 treatment was found to decrease ROS levels in SD rat myocardial tissue, suggesting that its antifibrotic effects may be partially attributed to its antioxidant properties [103].

**FIGURE 7:**
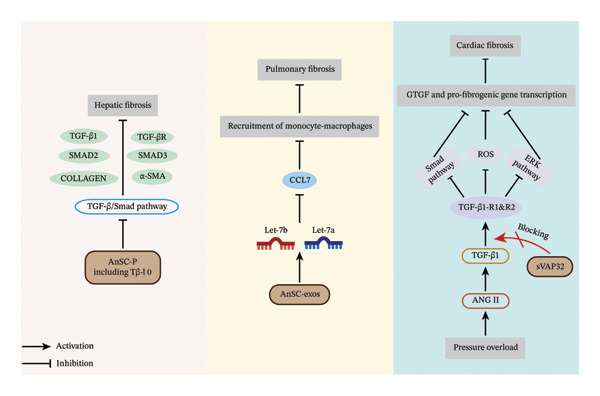
Antifibrotic effects of velvet antler and its active components across multiple organ systems. *Source:* [101–103].

Collectively, velvet antler and its bioactive components demonstrate significant antifibrotic effects in various organ fibrosis models. The underlying mechanisms include inhibition of the TGF‐β1/SMAD signaling pathway, reduction of ECM deposition, modulation of macrophage recruitment, and attenuation of oxidative stress (Figure 7). While extensive in vitro and in vivo studies support the antifibrotic potential of velvet antler, further research is needed to identify its precise active compounds, clarify its molecular targets, and assess its clinical applicability, thereby advancing its potential for use in fibrosis therapy.

### 4.8. Other Pharmacological Effects of Velvet Antler

Beyond the primary pharmacological activities outlined above, preclinical studies in vitro and in vivo have indicated that velvet antler and its extracts exhibit broad biological potential across various physiological functions. A summary of their functional mechanisms is provided in Table 2.

**TABLE 2:** Other pharmacological activities of velvet antler.

Categories	Mechanisms of effects of bioactive components				
	MW (kDa)	Sequence	Effects	Mechanisms	References
Protein			Protect LPS/d‐GalN‐induced liver injury in mice	↓MAPK, ↓NF‐κB	[10]
			Inhibition of APAP‐induced oxidative stress and apoptosis	↑Nrf2, ↓FoxO1	[11]
			Amelioration of ischemia‐hypoxia–induced endothelial cell injury in cardiac microvessels	↑PI3K/Akt	[104]
			Repair of UVB‐induced photodamage in HaCaT cells and ICR mice	↑MAPK, ↑TGF‐β/Smad	[105]
	65.53		Inhibits breast enlargement	↑Raf‐1/MEK/ERK	[106]
	22.589		Inhibits the growth of S180 solid tumors in tumor‐bearing mice	Mitochondria‐induced S‐phase cell cycle arrest	[107]

Peptide	3.2		Immunomodulatory effect on the immune system of mice	↑Th1, ↓Th2	[108]
	3.2		Amelioration of adriamycin‐induced myocardial injury	↑TGF‐β/SMAD	[7]
	7.0		Reduction of lithocholic acid–induced cholestatic liver injury	↓PI3K	[109]
			Neuroprotective effects against neurodegeneration in a Parkinson’s disease model	Inhibits oxidative stress and modulates gut microbiota	[26]
	0.73013	DNRYY	Significantly reduced blood pressure in a rat model of spontaneous hypertension		[81]
	7.2		Amelioration of carbon tetrachloride‐induced hepatotoxicity in mice	↓TLR/NF‐κB, ↓TGF‐β/Samd‐3	[110]
			Promoting chemosensitization and T‐cell infiltration in triple‐negative breast cancer with chemotherapy	↓TGF‐β 1	[93]
	7.2		Significant inhibition of lipopolysaccharide‐induced inflammatory factor production in myeloid cells		[111]
			Reduce ischemia/reperfusion‐induced cerebral ischemia	↑Nrf‐2/OH‐1, ↓NF‐κ B	[9]
			Attenuation of bleomycin‐induced pulmonary fibrosis in mice	↓ROCK/NF‐κB	[8]
			Amelioration of high‐fat diet–induced nonalcoholic fatty liver disease in rats	↑AMPK, ↓NF‐κB	[29]

Peptide monomer		LVLVEAELRE	Improve CUMS‐induced depression in mice	↓FGFR3 protein	[112]
	4500		Improve cognitive impairment in mice with Alzheimer’s disease	↓NLRP3	[113]
	5000		Inhibition of hypoxic brain damage in mice with middle cerebral artery blockade	Inhibits vascular endothelial cell injury and microglia inflammatory response	[114]

Collagen Type I	9 and 15		Promote bone marrow mesenchymal stem cell differentiation	↑ERK1/2, ↑p38‐MAPK	[115]

Nucleosides			Antifatigue effect		[14]

Korean Red Ginseng and Cervi Parvum Cornu			Improve FeCl_3_‐induced arterial thrombosis	↓ICAM‐1, ↓VCAM‐1	[116]

Polysaccharide			Inhibition of bone resorption in rats with high‐conversion osteoporosis	↑MAKP, ↑MMP‐9	[12]

In endocrine regulation, active proteins from velvet antler showed potential to inhibit mammary hyperplasia in a rat model of the condition [106]. Regarding tissue repair, velvet antler extract promoted hair growth by activating AKT and Wnt signaling pathways, stimulated proliferation of hair follicle stem cells in vitro, and delayed the telogen phase in the hair growth cycle of mice in a neurogenic alopecia model [117]. Furthermore, the potential value of velvet antler in preventing and treating neurological disorders has attracted attention. Available preclinical evidence suggests that velvet antler and its active components hold promise for applications such as antidepressant activity [112, 118], cognitive enhancement [113], and neuroprotection [26]. The underlying mechanisms may involve multifaceted regulatory effects, including modulation of neurotransmitter levels, enhancement of synaptic plasticity, promotion of neurotrophic factor expression (e.g., brain‐derived neurotrophic factor), and reduction of neural damage via inhibition of neuroinflammation. However, whether these mechanisms produce consistent effects in the human nervous system requires further investigation.

Regarding metabolic regulation, PAP has been found to improve nonalcoholic fatty liver disease induced by a high‐fat diet in rats [29], suggesting its potential application in metabolic disease interventions. Additionally, velvet antler exhibits positive effects on skin health. Research indicates that PAP alleviates UV‐induced skin damage in mice by mitigating oxidative stress and suppressing inflammation via the MAPK and TGF‐β/Smad signaling pathways [105].

In the cardiovascular system, velvet antler has demonstrated the ability to alleviate arterial thrombosis [116] and may exert vasoprotective effects by improving endothelial function [104]. Furthermore, its immunomodulatory properties have attracted considerable interest [108, 119]. It has been reported to influence immune homeostasis through regulation of immune cell activity and modulation of inflammatory cytokine expression, collectively contributing to overall health.

Currently, most potential biological effects of velvet antler are based on preclinical research and lack sufficient clinical validation. Future targeted clinical studies are needed to clarify its actual effects and application value in humans.

## 5. Safety, Quality Control, and Regulatory Challenges in Velvet Antler–Based Products

Velvet antler, a traditional Chinese medicinal material, has been used in China for thousands of years and is also widely consumed as a dietary supplement in international markets such as the United States [120]. Based on long‐term clinical use, velvet antler and its extracts are generally considered safe, with no major adverse effects reported to date. However, this widespread perception of safety has led research to focus predominantly on its pharmacological effects, resulting in a notable lack of studies on potential adverse reactions and toxicity. Systematic clinical safety assessment remains underdeveloped.

Regarding safety risks, toxicological studies on velvet antler and its extracts are still limited, especially concerning potential adverse effects in humans. Although animal toxicity studies suggest good safety at conventional doses (e.g., LD_50_ > 2000 mg/kg), in vitro experiments have shown cytotoxic effects of velvet antler at concentrations of 500 and 1000 μg/mL on HT22 hippocampal cells, RAW264.7 macrophages, and BV2 microglia [121]. Thus, further studies in animals and humans are needed to establish a safe dosage range. Notably, although clinical reports are extremely scarce, one case of drug‐induced liver injury (DILI) associated with velvet antler supplementation has been documented: a male with a history of testosterone‐related liver injury presented with significantly elevated liver enzymes (AST 360 U/L, ALT 1297 U/L) and acute liver injury symptoms after taking velvet antler supplements [122]. His liver markers gradually normalized following discontinuation. It should be noted that DILI is one of the most common reasons for regulatory action by the U.S. Food and Drug Administration (FDA) against drugs and dietary supplements. In traditional Chinese medicine, velvet antler is also used with caution, particularly in certain pathological conditions. Moreover, bioactive proteins or peptides in velvet antler may carry sensitization risks. Protein compounds are recognized as the main source of most food allergies [123], while low–molecular‐weight bioactive peptides generated via hydrolysis may reduce allergenic potential [124]. Although bioactive peptides generally exhibit fewer side effects, their safety still requires investigation due to possible retention of allergenic epitopes from parent proteins [123]. In summary, the safety of velvet antler in specific populations (e.g., children, pregnant women, and hormone‐sensitive patients) warrants further study. Significant challenges remain regarding the safety and quality consistency of its clinical application.

Inadequate quality control and regulatory gaps further complicate these safety concerns. Velvet antler is susceptible to contamination by exogenous substances during farming, processing, and storage. According to the ChP, animal‐derived medicines are characterized by complex compositions, perishability, and vulnerability to contamination by heavy metals, pesticide residues, and veterinary drug residues. Although the ChP requires testing for heavy metals such as lead (Pb), cadmium (Cd), arsenic (As), mercury (Hg), and copper (Cu), specific limits for heavy metals in animal‐derived medicines are not as strictly defined as for botanical drugs. While some studies on velvet antler powder or extracts have reported levels of toxic elements and microbial contaminants below the limits set by ASEAN guidelines [41, 121], systematic and continuous control of heavy metal and microbial contamination in velvet antler products remains a major challenge in the context of large‐scale industrialization and market distribution. This highlights the urgent need to establish a standardized quality monitoring system covering the entire supply chain, along with strengthened regulatory oversight.

Currently, velvet antler faces inconsistent regulatory classifications internationally. In different countries or regions, it may be categorized as a dietary supplement, traditional medicine, or modern drug. These regulatory discrepancies result in the absence of globally harmonized standards for pathogen inactivation and functional component testing. At the same time, the lack of standardized protocols, combined with the limited adoption of key antiadulteration technologies—such as DNA barcoding and Raman spectroscopy—within the actual industry chain, collectively constitutes the main barrier to standardizing velvet antler products and advancing their translation into modern biopharmaceutical applications.

Therefore, future efforts should prioritize establishing a systematic safety assessment framework for velvet antler, refining quality control standards, and promoting its evidence‐based standardization and modern application.

## 6. Conclusion and Future Perspectives

Velvet antler, a high‐value medicinal and food dual‐use material in traditional Chinese medicine, is rich in characteristic bioactive components such as amino acids, peptides, and nucleosides. It exhibits a wide range of beneficial biological activities, including wound repair, antioxidant, anti‐inflammatory, and antifatigue effects. Globally, numerous velvet antler‐based foods and dietary supplements have been developed for nutritional support and as adjunctive therapies for various conditions. Although basic research and identification techniques have made phased progress, several core challenges persist in the industrialization and clinical translation of velvet antler.

First, quality control and safety remain primary bottlenecks. As an animal‐derived material, velvet antler is susceptible to influences from breeding conditions and feed, posing potential risks of contamination with heavy metals and pesticide residues. Furthermore, existing research lacks systematic evaluation of its potential long‐term side effects. Future efforts should establish standardized clinical trials to determine safety thresholds and develop targeted risk‐assessment systems, particularly refining applicable standards for its use as a functional food.

Second, raw material standardization remains inadequate. Significant variations in velvet antler composition arise from differences in species, growth stages, and processing methods, compounded by the absence of unified quality evaluation metrics and production standards. There is an urgent need to establish a standardized system covering the entire chain—from farming and processing to distribution—that quantifies core active ingredient content and dose–response relationships to ensure product consistency.

Third, industrialization faces technical barriers. Laboratory‐level identification techniques (e.g., multiomics) and active‐ingredient extraction processes are often not directly scalable. Cost‐effective, high‐efficiency industrial‐grade technologies must be developed to address critical challenges such as raw material supply stability and production process optimization.

Furthermore, most pharmacological and compositional studies on velvet antler rely on in vitro cell models or animal experiments, which cannot fully replicate the complexity of human physiology. The metabolic pathways of active components in vivo and the regulatory roles of key enzymes remain unclear. Moving forward, more human clinical trials are needed to validate preclinical findings. Concurrently, further research is required to elucidate the structure–activity relationships, synergistic mechanisms, and quantitative dose–response correlations of core active components such as glutamic acid and velvet antler peptides.

Ongoing developments in velvet antler research may broaden its application in both the food and pharmaceutical sectors. Beyond applications in precision nutrition, personalized health products, functional foods, and biopharmaceuticals, there is an urgent need to expand its use in specialized health areas such as neuroprotection and antifibrosis. Furthermore, a deeper understanding of the digestive behavior, release kinetics, and bioavailability of velvet antler’s active components within complex food systems will facilitate the design of high–value‐added products, thereby promoting the modernization and internationalization of traditional Chinese medicine.

## Author Contributions

Conceptualization: Xiaopeng Liu and Ning Jiang; methodology: Xiaopeng Liu and Ning Jiang; formal analysis: Xiaopeng Liu, Ning Jiang, and Maodie Zhang; data curation: Xiaopeng Liu, Ning Jiang, and Maodie Zhang; writing–original draft preparation: Ning Jiang and Maodie Zhang; writing–review and editing: Xiaopeng Liu, Ning Jiang, and Maodie Zhang.

## Funding

This work was supported by the National Natural Science Foundation of China under Grant No. 82260754.

## Disclosure

All authors have read and agreed to the published version of the manuscript.

## Conflicts of Interest

The authors declare no conflicts of interest.

## Data Availability

The data that support the findings of this study are available from the corresponding authors upon reasonable request.
